# Clinical outcomes of trabecular microbypass stent (iStent) implantation in medically controlled open-angle glaucoma in the Korean population

**DOI:** 10.1097/MD.0000000000021729

**Published:** 2020-08-14

**Authors:** Hee Jun Kim, Su-Ho Lim

**Affiliations:** a Good Doctors Eye Hospital, Ulsan; b Department of Ophthalmology, Yeungnam University College of Medicine, Daegu; c Department of Ophthalmology, Daegu Veterans Health Service Medical Center, Daegu, Republic of Korea.

**Keywords:** glaucoma, MIGS, POAG, surgery, trabecular microbypass stent

## Abstract

To evaluate the safety and efficacy [intraocular pressure (IOP)-lowering effect and medication use] of a single trabecular microbypass stent (iStent; Glaukos Corp, San Clemente, CA) for medically controlled open-angle glaucoma.

This retrospective case series included 42 eyes of 34 patients with medically controlled open-angle glaucoma with IOP less than 21 mm Hg. Clinical outcomes analyzed were IOP, medication use, corrected distance visual acuity (CDVA), and surgical complications. Surgical success was defined according to 4 criteria: IOP < 21 mm Hg without medication; IOP < 18 mm Hg without medication; IOP < 15 mm Hg without medications; and IOP < 18 mm Hg with or without medication. Patients were followed for a minimum of 6 months postoperatively.

Mean IOP was reduced from 15.8 ± 2.8 mm Hg to 14.5 ± 2.8 mm Hg (*P* < .001), while mean number of medications decreased from 2.2 ± 1.2 to 0.8 ± 1.1 at final visit (*P* < .001). Surgical success rates were 78.6%, 61.9%, 57.1%, and 97.6% at 6 months and 78.6%, 59.5%, 52.4%, and 95.2% at final visits according to criteria A, B, C, and D. Meanwhile, 59.5% of patients were medication-free at their final visit. The relative risk of surgical failure by Criteria B and C was 4.337 (95% confidence interval: 1.799–10.454) and 3.717 (95% confidence interval: 1.516–9.116) times greater in the higher-medication group (3 or more preoperative medications), respectively. CDVA was significantly improved from 0.41 ± 0.10 to 0.09 ± 0.07 LogMAR in the combined phacoemulsification and iStent implantation group (*P* < .001). There was no case whose vision was threatened (vision loss of 2 or more lines) or who showed severe complications after surgery.

Single trabecular microbypass stent implantation was effective in reducing IOP and medication usage in patients with open-angle glaucoma with a low preoperative IOP. Our results imply that it is more difficult to achieve low target IOP control in eyes with higher numbers of preoperative medications.

## 1 Introduction

Glaucoma is one of the leading causes of blindness worldwide.^[1,2]^ The goal of glaucoma treatment is to maintain the patient's visual function and quality of life at a sustainable cost.^[3]^ Many previous studies have sought to lower elevated intraocular pressure (IOP), which is widely established as the primary modifiable risk factor for both “onset” and “progression” of glaucoma.^[4,5]^ Thus, the ideal treatment for glaucoma should encompass continuous IOP management along with a favorable safety profile.^[5,6]^

Although filtration surgery such as trabeculectomy or glaucoma drainage device implantation are effective for IOP lowering, they exhibit the possibility of serious complications including hypotony, inflammation, reoperation, hyphema, bleb-related complications, and loss of vision in the Tube versus Trabeculectomy study.^[7]^ In this context, the first course of treatment is typically ocular hypotensive mediations.^[8]^ However, these medications had some limitations including compliance, tolerability, and conjunctival toxicity, which might affect the outcomes of surgery, ocular allergy, and secondary ocular surface diseases such as corneal epitheliopathy.^[9,10]^ Thus, patients have often been advised to undergo watchful waiting until a certain amount of glaucomatous damage produced a more acceptable risk-to-benefit profile of traditional surgeries.^[4]^

The large number of new glaucoma drainage devices that have emerged in recent years is a testament to the desire to find a “safe and simple” surgical procedure to treat glaucoma.^[11]^ As a result, the rapid influx of new devices onto the market has caused some to wonder whether we are entering a “new era of microstent surgery” in glaucoma management.^[4,6,8,11]^ The iStent (Glaukos Corp, San Clemente, CA), a trabecular microbypass stent, is the first implant approved by the United States (US) Food and Drug Administration for use during microinvasive glaucoma surgery.^[8,12]^ Most previous studies considering the efficacy of trabecular microbypass stenting included patients with “uncontrolled” IOP or a mean medicated IOP greater than 21 mm Hg,^[4,6,8,13]^ while there are limited reports of outcomes after phaco-combined surgery in “controlled” glaucoma.^[12]^ Moreover, to our knowledge, there has been no study comparing outcomes according to preoperative number of medications used in the Korean population.

In this study, we sought to analyze outcomes after single trabecular microbypass stent implantation in medically controlled open-angle glaucoma (OAG); compare outcomes according to preoperative number of medications; and compare outcomes between combined phacoemulsification and iStent implantation (Combo group) and stand-alone iStent implantation (Solo group).

## 2 Methods

### 2.1 Patients

This was a single-center, retrospective case series conducted at the Daegu Veterans Health Service Medical Center from January 2018 to December 2019. The study protocol was approved by the Institutional Review Board of Daegu Veterans Hospital (2020-10). All participants provided signed informed consent, and this study adhered to the tenets of the Declaration of Helsinki.

**Figure 1:**
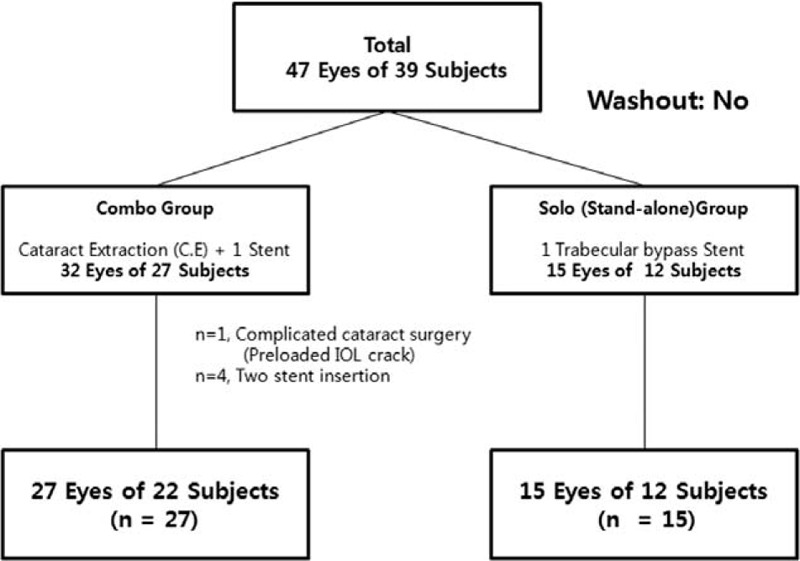
Patient enrollment flow-chart.

The respective study groups included eyes with visually significant cataract and coexisting OAG undergoing combined cataract extraction (Combo group) or eyes with OAG with poor compliance, drug allergy, or need to reduce ocular hypotensive medications (Solo group). Medically controlled OAG was defined as cases where the medicated IOP was below 21 mm Hg and baseline unmedicated IOP was 21 mm Hg or above with corrected central cornea thickness. Meanwhile, the following were excluded: eyes with less than 6 months of follow-up; eyes undergoing another procedure during the operation; eyes with angle-closure glaucoma or secondary glaucoma such as pseudoexfoliative glaucoma, pigmentary glaucoma, or neovascular glaucoma; or eyes with a history of previous glaucoma surgery or laser treatment for glaucoma such as argon laser trabeculoplasty or selective laser trabeculoplasty. Figure 1 presents a flowchart of patient accountability through 24 months.

### 2.2 Ophthalmic examination

All subjects underwent complete ophthalmic examinations, including best-corrected visual acuity measurement, Goldmann applanation tonometry, slit-lamp biomicroscopy, gonioscopy using a Goldmann 3-mirror lens, dilated fundus examination, and retinal nerve fiber layer measurements using spectral-domain optical coherence tomography (RT-Vue 100; RT Vue Inc, Fremont, CA). The margins of the optic cup were defined as the point of maximal inflection of vessels crossing the neuroretinal rim. The vertical cup diameter was measured as the vertical distance between the points of maximum centrifugal extension of the cup between 11 and 1 o’clock and between 5 and 7 o’clock by a single glaucoma specialist (S-HL). Automated perimetry was performed using a Humphrey Visual Field Analyzer 740i (Carl Zeiss Meditec Inc, Dublin, CA) and the 24-2 Swedish Interactive Threshold Algorithm. Visual field (VF) defects were defined as follows: glaucomatous VF defects corresponding to optic nerve head or retinal nerve fiber layer changes; glaucoma hemifield test results outside of the normal limits; and a cluster of three or more nonedge, contiguous points not crossing the horizontal meridian, with *P* values < .05 compared with the age-matched normal on the pattern deviation plot, one of which must have a *P* value < .01.^[14]^ Finally, Hodapp–Anderson criteria and the enhanced glaucoma staging system were applied to classify the severity of glaucoma.^[15]^

### 2.3 Surgery

All surgeries were performed by 1 glaucoma specialist (S-HL) who has completed a wet-lab training program and glaucoma fellowship. Study subjects underwent implantation of 1 iStent trabecular microbypass stent from January 2018 to June 2019. Each single-piece, heparin-coated, titanium stent had a length of 1.0 mm, height of 0.33 mm, a snorkel bore diameter of 120 μm.^[4]^ Briefly, under topical anesthesia using a 0.5% proparacaine eye drop, phacoemulsification was completed through a temporal clear incision in the Combo group. An ophthalmic viscosurgical device was placed in the anterior chamber to deepen the angle and visualize the trabecular meshwork. A single trabecular microbypass stent was inserted into Schlemm canal (SC) nasally (3 to 4 o’clock in the right eye vs. 8 to 9 o’clock in the left eye) using a Swan Jacob gonioprism. Removal of the ophthalmic viscosurgical device was completed, and the eye was filled with a balanced salt solution.^[8,12]^

### 2.4 Postoperative medications and follow-up

Postoperatively, topical 0.5% gatifloxacin and 0.1% fluorometholone acetate eye drops were prescribed 4 times daily for 1 week and then tapered according to inflammation resolution. In accordance with the World Glaucoma Association's guidelines,^[16]^ each patient's best-corrected visual acuity, IOP, glaucoma medication use, and complications were recorded for 1 day, 1 week, 1 month, 2 months, 3 months, 6 months, and 1 year postoperatively. Patients with less than 6 months of follow-up after surgery were excluded from analysis. Postoperatively, glaucoma medications were added per the surgeon's opinion based on disease severity and target IOP. In general, a glaucoma medication was started if the IOP exceeded 18 mm Hg or in the case of concerning optic nerve or VF changes given the landmark study of Advanced Glaucoma Intervention Study demonstrated that VF progression is delayed when IOP is consistently maintained below 18 mm Hg.^[17]^

### 2.5 Data analysis

Surgical successes were defined according to 4 criteria: IOP < 21 mm Hg without medication; IOP < 18 mm Hg without medication; IOP < 15 mm Hg without medication; and IOP < 18 mm Hg with or without medication. Patients were followed for a minimum of 6 months postoperatively.

Statistical analyses were conducted using the Statistical Package for the Social Sciences for Windows software version 18.0 (IBM Corp, Armonk, NY). The Mann–Whitney test and *χ*^2^ tests were used to compare baseline characteristics between the Combo and Solo groups. Repeated-measures analysis of variance (ANOVA) was used to compare the IOP and number of medications used during the study period. A *P* value < .05 was defined as statistical significance.

## 3 Results

### 3.1 Baseline characteristics

Table 1 compares the baseline characteristics of the Combo and Solo groups. The mean age was 73.7 ± 4.5 years in the Combo group and 73.7 ± 3.7 years in the Solo group (*P* = .577). The mean IOP was 15.8 ± 2.8 mm Hg, and there was no difference in preoperative IOP between the groups (*P* = .860). However, the number of medications in the Solo group was higher than that in the Combo group (3.0 ± 1.1 vs 1.8 ± 1.0; *P* = .005). Although the MD, PSD, and VF index demonstrated slightly lower values in the Solo group, there were no significant differences in these parameters between the 2 groups.

**Table 1:**
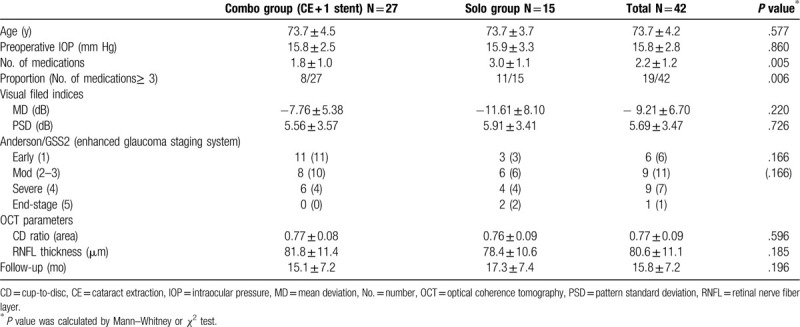
Demographic and baseline characteristics of the study population.

### 3.2 IOP reduction during 6 months in the intent-to-treat population

**Figure 2:**
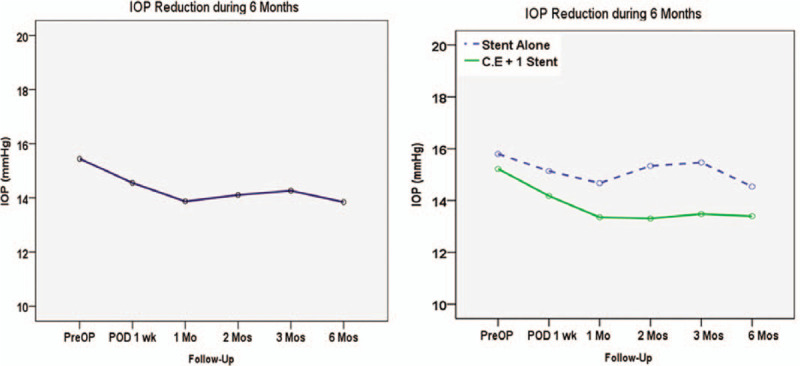
IOP reduction over 6 mo in the intent-to-treat population. At 6 mo, IOP was reduced from 15.8 ± 2.8 mm Hg to 13.9 ± 2.1 mm Hg (A, *P* < .001). The number of medications decreased from 2.24 ± 1.20 to 0.73 ± 1.07 (*P* < .001). In the combo group, IOP decreased from 15.8 ± 2.5 mm Hg on a mean of 1.8 medications to 13.5 ± 1.4 mm Hg on a mean of 0.3 medications. In the solo group, IOP decreased from 15.9 ± 3.3 mm Hg on a mean of 3.0 medications to 14.5 ± 2.8 mm Hg on a mean of 1.5 medications (B). IOP = intraocular pressure.

In the whole study group, the mean IOP and number of medications decreased from 15.8 ± 2.8 mm Hg to 13.9 ± 2.1 mm Hg and from 2.2 ± 1.2 to 0.7 ± 1.0 at 6 months, respectively (*P* < .001 and *P* < .001). In the Combo group, the mean IOP decreased from 15.8 ± 2.5 mm Hg on a mean of 1.8 ± 1.0 medications to 13.5 ± 1.4 mm Hg on a mean of 0.3 ± 0.7 medications at 6 months. In the Solo (stand-alone) group, mean IOP decreased from 15.9 ± 3.3 mm Hg on a mean of 3.0 ± 1.1 medications to 14.5 ± 2.8 mm Hg on a mean of 1.5 ± 1.0 medications at 6 months. At 6 months, the mean number of medications used was still lower in the Combo group than in the Solo group. However, the reduction in medication count did not show a statistically significant difference between the 2 groups (*P* = .673) (Fig. 2).

### 3.3 Proportional analysis of IOP reduction

Table 2 indicates patients achieving IOP reduction at 6 months and final visits in both groups. At 6 months, the proportion of patients adhering to criterion A was 78.6% (IOP < 21 mm Hg without medication), that for criterion B was 61.9% (IOP < 18 mm Hg without medication), that for criterion C was 57.1% (IOP < 15 mm Hg without medication), and that for criterion D was 97.6% (IOP < 18 mm Hg with or without medication). Meanwhile, 59.5% of patients were medication-free at their final visits.

**Table 2:**
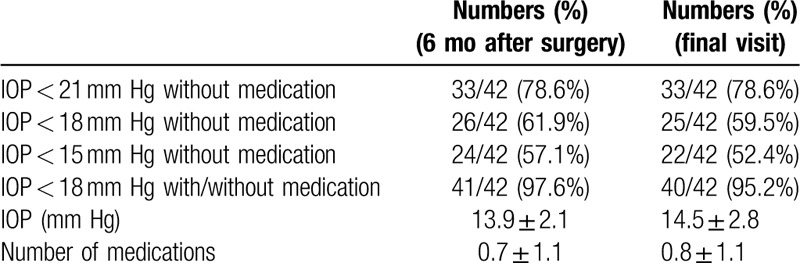
Intraocular pressure reduction at 6 mo and at the final visit in the intent-to-treat population.

Table 3 showed the postoperative outcomes according to the preoperative number of medications. Considering complete success by criterion B, the eyes with higher preoperative medication demonstrated poorer IOP control. The relative risk of surgical failure by criteria B was 4.337 times greater (95% confidence interval: 1.799–10.454; *P* < .001) in the higher preoperative medication group (using 3 or more medications). Moreover, the relative risk of surgical failure based on criterion C was 3.717 times greater (95% confidence interval: 1.516–9.116; *P* < .001) in the higher medication group.

**Table 3:**
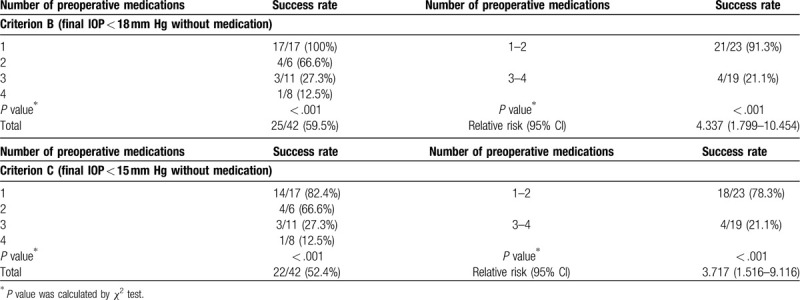
Postoperative outcomes according to preoperative number of medications.

### 3.4 Visual acuity

The corrected distance visual acuity was significantly improved from 0.41 ± 0.10 to 0.09 ± 0.07 LogMAR in the Combo group. There was no case of refractive surprise or vision loss over 1 month in either group.

### 3.5 Postoperative ocular complications

**Figure 3:**
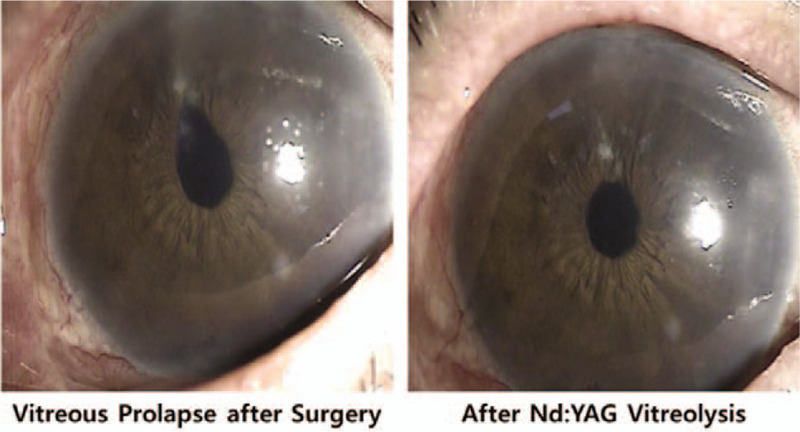
A case with vitreous prolapse after trabecular microbypass stent implantation. After Nd:YAG laser treatment, vitreous prolapse resolved without complications.

**Figure 4:**
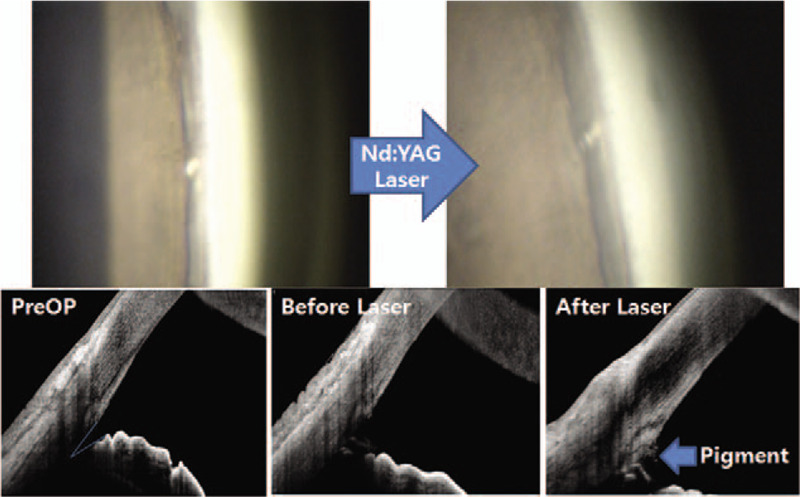
A case with stent obstruction. Stent obstruction was deemed mild, and resolved after Nd:YAG laser treatment.

Most ocular complications occurred in the early postoperative period. Table 4 reveals the frequency of complications. The most frequently reported side effects in order were transient IOP spike > 25 mm Hg, hyphema, anterior chamber reaction, vitreous prolapse, and stent obstruction. Vitreous prolapse was resolved after neodymium-doped yttrium aluminum garnet laser application (Fig. 3). In 1 eye, stent obstruction was deemed mild and ultimately resolved after neodymium-doped yttrium aluminum garnet laser treatment (Fig. 4).

**Table 4:**
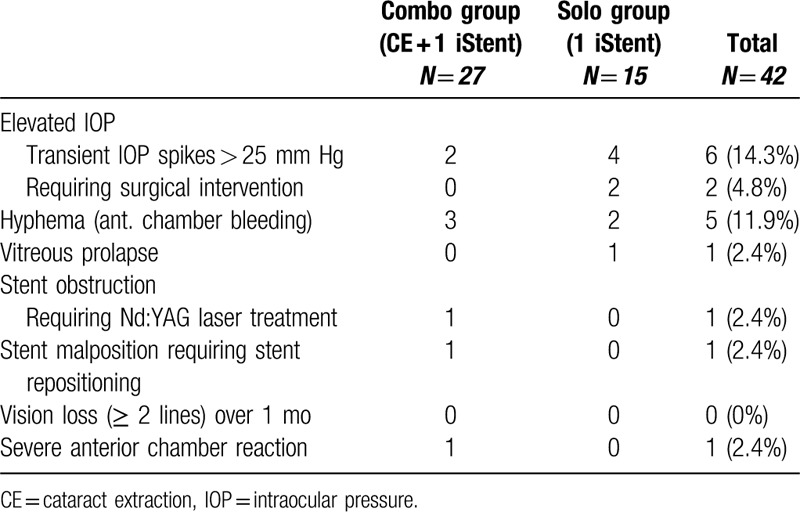
Frequency of reported postoperative complications.

### 3.6 Secondary surgical interventions

**Figure 5:**
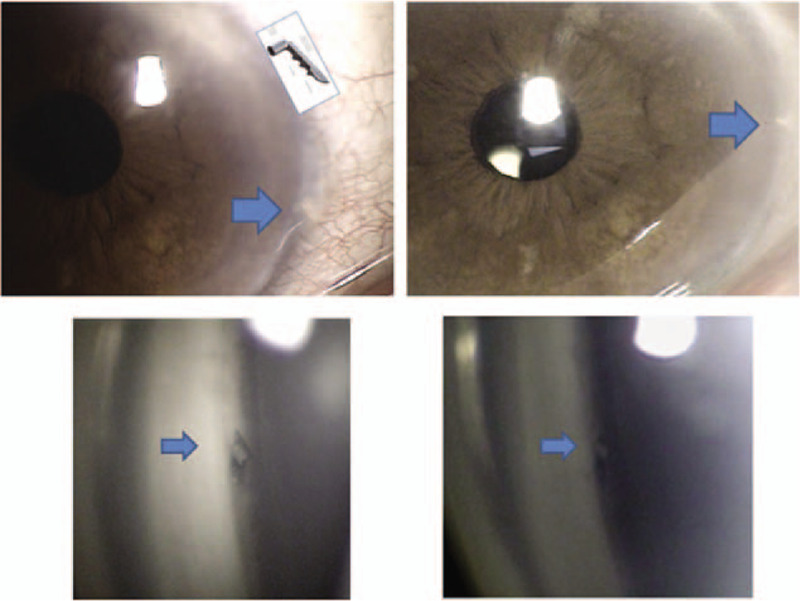
A case with trabecular microbypass stent malposition. After repositioning of the iStent, the patient showed favorable IOP control under 15 mm Hg.

One patient in the Combo group required stent repositioning (Fig. 5). After repositioning of the iStent, he showed good IOP control at less than 15 mm Hg. Two patients received additional surgical interventions including 1 trabeculectomy and 1 Ahmed valve implantation due to poor IOP control.

## 4 Discussion

In the present study, single trabecular microbypass stent implantation demonstrated effectiveness in reducing IOP and medication usage in patients with OAG with a low preoperative IOP. Of note, the reduction in medication count did not show a statistically significant difference between the 2 study groups (Combo group vs Solo group). Our study suggests a mean additional IOP reduction of 1.8 mm Hg (12%) is attainable without a wash-out period, with 78.6% of eyes maintaining an IOP < 21 mm Hg, 59.5% of eyes maintaining an IOP < 18 mm Hg without medication, 52.4% of eyes maintaining an IOP < 15 mm Hg without medication, and 95.2% of eyes maintaining an IOP < 18 mm Hg with or without medication during a mean 15.8 months of follow-up. Similarly, Neuhann and Neuhann^[18]^ reported that 96.3% of eyes had an IOP ≤ 18 mm Hg, 58.5% of eyes had an IOP ≤ 15 mm Hg, and 81.1% of eyes were free of any medication at 12 months after surgery for iStent inject. These findings are similar to those of previous studies.^[4,8,19,20]^

In the Combo group, previous randomized controlled trial studies in the United States by Craven et al demonstrated that a statistically significant 72% of patients who received combination surgery maintained an IOP ≤ 21 mm Hg at 12 months and 61% did so at 24 months.^[8]^ A smaller independent randomized controlled trial by Fea^[19]^ showed similar results with a reduction in ocular hypotensive medications for up to 4 years.^[20]^ Other relevant studies are summarized in Table 5.^[4,8,13,20–22]^ In solo group, Katz et al ^[4]^ reported prospective randomized results following the stand-alone iStent procedure in multiple countries (USA, Italy, Germany, and Spain). These authors included a total of 119 subjects stratified as 38 single-stent, 41 two-stent, and 40 three-stent cases. In their study, the mean IOP was 13.5 mm Hg at 6 months, 14.9 mm Hg at 12 months, and 15.0 mm Hg at final visits. An IOP reduction ≥ 20% without medication was achieved in 89% of study participants at 12 months. However, the need for additional medication increased in the single-stent group [4/38 eyes at month 12 and 18/38 eyes (52.6%) at month 42]. In our research, the Combo group showed an IOP decrease from 15.8 mm Hg to 13.5 mm Hg at 6 months and the Solo group showed an IOP decrease from 15.9 mm Hg to 14.5 mm Hg. According to previous studies and our results, we concluded that the combination of phacoemulsification and iStent implantation or iStent implantation alone both can reduce IOP and medication burden in glaucoma patients.^[4,8,12,13,20]^

**Table 5:**
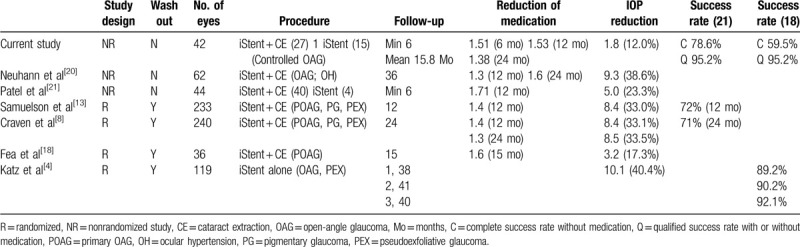
Comparison of outcomes after trabeulcar microbypass stent implantation with previous studies.

However, most previous studies assessing the efficacy of trabecular microbypass stent use included patients with uncontrolled IOP or a mean medicated IOP higher than 21 mm Hg,^[4,6,13]^ while more limited studies reported outcomes after combined-phaco surgery in controlled glaucoma.^[12]^ Seibold et al ^[12]^ reported outcomes after a combination procedure (phacoemulsification and iStent implantation) in controlled OAG. According to their findings, mean IOP decreased from 14.7 mm Hg to 13.2 mm Hg, and mean medication count decreased from 1.81 to 1.41. These findings are quite similar to our results of a reduction in IOP from 15.8 mm Hg to 13.9 mm Hg at final visits.

The majority of OAG cases in Korea^[23,24]^ and Japan^[25]^ are composed of low-tension glaucoma or normal-tension glaucoma; thus, our results seem to be important in management of glaucoma in the Asia-Pacific area. As it is, in these populations, the main drive for implantation of the microbypass stent was to reduce dependence on medications.^[12]^ In this context, we should consider not only IOP, but also number of preoperative medications. However, to our knowledge, there is no published article comparing results according to number of preoperative medications. To the best of our knowledge, this study is the first to compare results according to number of preoperative medications. Interestingly, the complete success rates by criterion B (IOP < 18 mm Hg without medication) were 100.0%, 66.6%, 27.3%, 12.5% for groups (1, 2, 3, and 4), respectively. Moreover, the lower-medication group (2 or less medications taken) presented a higher success rate than the higher-medication group (3 or 4 medications taken) retrospectively. The relative risk of surgical failure was 4.337 (95% confidence interval: 1.799–10.454) times greater in the higher-medication group. A similar pattern was observed based on criterion C (IOP < 15 mm Hg without medication). The respective complete success rate by criterion C was 82.4%, 66.6%, 27.3%, and 12.5% for groups (1, 2, 3, and 4). The relative risk of surgical failure was 3.717 (95% confidence interval: 1.516–9.116; *P* < .001) in the higher medication group based on criterion C.

Our results raise the question of the reason for higher surgical failure in the higher-medication group (3 or more medications) and the clinical relevance of these findings. The iStent implantation enhances the physiologic outflow pathway in the context of normal posttrabecular pathways. Thus, the properties of SC including morphology, lymphatics-like features, and biomechanics of the SC and resistance of the posttrabecular pathway seem to be important in the IOP-lowering effect. First, the morphological abnormalities of SC in primary OAG (POAG) might affect the IOP-lowering effect. One histopathologic study demonstrated that the length of the SC in POAG patients was significantly shorter than that in normal-tension glaucoma.^[26]^ The elevation of IOP may compress the trabecular meshwork and result in a collapse of the SC, pathological steps that also increase outflow resistance and contribute to the pathobiology of glaucoma development.^[27]^ Second, some functional characteristics, such as absence of pericyte coverage, a discontinuous basement membrane, and absence of blood filling, indicate that SC shares traits with the lymphatic vasculature. As a result, lymphatic defects caused ocular hypertension and glaucoma in a mouse model.^[28]^ Third, we also consider the biomechanics of the SC. In SC cells, vacuole and pore formation are dependent on pressure and most likely related to tissue stiffness. Overby et al^[29]^ reported that pore formation correlated with the stiffness of the subcortical cytoskeleton in SC cells and noted that glaucomatous SC cells exhibited both a stiffer subcortical cytoskeleton and a reduced ability to form pores. These observations suggest that some drugs that directly or indirectly modify the cytoskeleton may decrease cell stiffness and result in reduced outflow resistance.^[30,31]^

Given these findings, greater medication usage or higher preoperative IOP might lead the poor outcomes after trabecular microbypass stent implantation. Thus, this study indicates that it is more difficult to achieve low target IOP control in eyes with higher numbers of preoperative medications. Thus, during the informed consent process, ophthalmologists should consider the clinical benefit-to-risk of IOP-lowering or a decrease in medication use.^[7,12,17]^

Our study has several limitations. First, the number of participants was relatively small, and the study adopted a retrospective design without wash-out periods. Second, phacoemulsification itself might strengthen the IOP-lowering effects shown by the OHTS study.^[32]^ Third, our study is limited to up to 2 years of follow-up. Thus, further prospective studies with larger numbers of patients are needed to verify risk factors for surgical failure. Despite these limitations, however, this study is the first to compare outcomes according to number of preoperative medicines and results from medically controlled OAG in the Korean population.

In conclusion, combined phacoemulsification and iStent implantation (Combo procedure) or iStent implantation alone (Solo procedure) seem to be effective in lowering the IOP in medically controlled glaucoma. The overall efficacy of IOP reduction in patients showed similar results in previous publications.^[8,12,20]^ Managing IOP to a target IOP is common in clinical practice even in low-IOP cases on medication. Thus, the potential to achieve a target IOP with a lower medication burden is important in management of glaucoma.^[8]^ Our study added that the relative risk of surgical failure seems to be related to a higher preoperative medication count. Thus, trabecular microbypass stent implantation is recommended more strongly in cases of mild to moderate glaucoma using fewer preoperative medications.

## Acknowledgment

The authors thank eWorldEditing, Inc for providing the editing services.

## Author contributions

**Conceptualization:** Hee Jun Kim and Su-Ho Lim.

**Data Curation:** Hee Jun Kim, Su-Ho Lim.

**Methodology:** Su-Ho Lim.

**Supervision:** Su-Ho Lim.

**Surgery:** Su-Ho Lim.

**Validation:** Hee Jun Kim, Su-Ho Lim.

**Writing and review:** Hee Jun Kim, Su-Ho Lim.

## Corrections

When originally published, the grant number and funding recipient for the VHS Medical Center Research Grant was not included and has since been added to the footnote details.
